# 2,3,4-Trihydroxybenzophenone Disassembles Amyloid β Aggregates and Ameliorates Synaptic Deficits

**DOI:** 10.3390/pharmaceutics18030320

**Published:** 2026-03-02

**Authors:** Eunbi Cho, Kumju Youn, Huiyoung Kwon, Ho Jung Bae, Minho Moon, Mira Jun, Dong Hyun Kim

**Affiliations:** 1 Department of Pharmacology, College of Medicine, Konkuk University, Chungju 27478, Republic of Korea; bee2634@naver.com; 2 Korean Medicine-Application Center, Korea Institute of Oriental Medicine, Daegu 41062, Republic of Korea; 3 Department of Food Science and Nutrition, Dong-A University, 37 Nakdong-daero 550 beon-gil, Saha-gu, Busan 49315, Republic of Korea; kjyoun@dau.ac.kr (K.Y.); mjun@dau.ac.kr (M.J.); 4 Global Institute for Advanced Nanoscience & Technology (GIANT), Changwon National Univerisy, Changwon 51140, Republic of Korea; kwonhuiyoung@naver.com (H.K.); baehj321@changwon.ac.kr (H.J.B.); 5 School of Interdisciplinary Natural Science with Flexible Major, Golcal Advanced Institute of Science and Technology, Chanwon National University, Changwon 51140, Republic of Korea; 6 Department of Biochemistry, College of Medicine, Konyang University, Daejeon 35365, Republic of Korea; hominmoon@konyang.ac.kr; 7 Research Institute of Medical Science, Konkuk University School of Medicine, 268 Chungwon-daero, Chungju 27478, Republic of Korea

**Keywords:** 2,3,4-tetrahydrobenzophenone, amyloid β, aggregation, Alzheimer’s disease, synaptic plasticity, 5XFAD

## Abstract

**Background/Objectives**: Alzheimer’s disease (AD) is a progressive neurodegenerative disorder for which no disease-modifying therapy that halts or substantially slows disease progression is currently available. Although antibody therapies targeting amyloid β (Aβ) have recently received FDA approval, their high cost, limited efficacy, and potential adverse effects highlight the need for alternative solutions. Therefore, the development of low-molecular-weight compounds capable of reducing toxic Aβ aggregates is of considerable interest. In this study, we investigated the effects of 2,3,4-trihydroxybenzophenone (THB) on the inhibition and disassembly of Aβ_1–42_ aggregates through in vitro and in vivo experiments. **Methods**: In vitro assays were performed to evaluate the effects of THB on Aβ_1–42_ aggregation and fibril disassembly. Cell viability assays and hippocampal slice electrophysiology were conducted to assess neurotoxicity and synaptic function. In vivo effects were examined in Aβ_1–42_ aggregate-injected mice and in 5 Familial AD mutations (5XFAD) mice using behavioral, histological, and electrophysiological analyses. **Results**: THB inhibited Aβ_1–42_ aggregation in a concentration-dependent manner and promoted the disassembly of preformed fibrils. THB attenuated Aβ_1–42_-induced Neuro2a cell death and restored Aβ_1–42_ aggregate-associated long-term potentiation (LTP) deficits in hippocampal slices. In Aβ_1–42_ aggregate-injected and 5XFAD mice, THB reduced amyloid pathology and neuroinflammatory markers and improved synaptic function and memory performance. **Conclusions**: These findings suggest that THB modulates pathogenic Aβ_1–42_ assemblies and provides a structural basis for the development of small-molecule modulators of Aβ_1–42_ aggregation with potential preventive or disease-modifying applications in AD.

## 1. Introduction

Alzheimer’s disease (AD) is the most common neurodegenerative disorder and accounts for approximately 50–80% of all dementia cases [1]. AD is characterized by progressive cognitive decline resulting from widespread neuronal damage, leading to emotional and social impairments [2,3]. The disease not only affects patients but also imposes a substantial burden on families and society.

Although the precise pathogenesis of AD remains incompletely understood, several pathological features have been consistently observed in affected brains, including amyloid β (Aβ) plaques, tau neurofibrillary tangles (NFTs), neuroinflammation, neuronal loss, synaptic dysfunction, and cholinergic deficits [4,5,6]. Despite extensive research, the development of effective disease-modifying therapies remains limited. Currently approved treatments include cholinesterase inhibitors (donepezil, rivastigmine, galantamine, tacrine), the N-methyl-D-aspartate (NMDA) receptor antagonist memantine, and monoclonal antibodies targeting Aβ species (aducanumab, donanemab and lecanemab) [7]. However, these treatments primarily provide symptomatic relief and may be associated with adverse effects, underscoring the need for alternative therapeutic strategies [8,9].

Abnormal aggregation of Aβ is associated with increased β-sheet content, a hallmark of AD pathology [10]. Aβ plaques initially accumulate in the entorhinal and frontal cortices, and plaque burden correlates with the severity of memory impairment [11]. Because Aβ aggregation is believed to begin years before the onset of clinical symptoms, early inhibition or removal of toxic aggregates may delay or prevent disease progression [12]. Given the limitations and safety concerns associated with antibody-based therapies, targeting Aβ aggregation using small-molecule modulators may represent a complementary and potentially more accessible strategy.

In the present study, we focused on the Aβ_1–42_ isoform, which exhibits greater aggregation propensity and neurotoxicity than Aβ_1–40_. 2,3,4-Trihydroxybenzophenone (THB) is a benzophenone derivative containing three hydroxyl groups and is classified as a polyphenolic compound. Previous studies have reported that THB inhibits Aβ aggregation and tau filament formation in vitro [13]; however, those studies primarily examined Aβ1–40 and did not evaluate in vivo efficacy. Therefore, we investigated whether THB inhibits and disassembles Aβ_1–42_ aggregates and whether these effects translate into improvements in synaptic function, neuroinflammation, and cognitive performance in AD models.

## 2. Materials and Methods

### 2.1. Materials

Aβ_1–42_ was obtained from Anaspec (Fremont, CA, USA), while THB, Thioflavin T (ThT), and Thioflavin S (ThS) were purchased from Sigma-Aldrich (St. Louis, MO, USA). For the immunological assays, goat anti-Iba-1 and rabbit anti-GFAP primary antibodies were purchased from Abcam (Cambridge, UK). To evaluate cellular viability and cytotoxicity, 3-(4,5-dimethylthiazol-2-yl)-2,5-diphenylatetrazolium bromide (MTT) and Cytotoxicity Detection Kit^PLUS^ (for LDH assay) were purchased from Sigma-Aldrich (St. Louis, MO, USA). All other reagents and supplementary chemicals were of analytical grade and sourced from standard commercial suppliers.

### 2.2. Animals

To investigate an Aβ_1–42_ aggregate-induced cognitive deficit, a total of 85 male CD-1 mice (6 weeks old, 25–30 g) were obtained from Samtako (Osan, Republic of Korea). 80 mice were used for behavioral tests, and 5 mice were used for electrophysiology. The animals were allowed a 7-day acclimatization period under standard laboratory conditions before the initiation of experiments at 7 weeks of age. Additionally, 5 Familial AD mutations (5XFAD) transgenic mice were sourced from the Jackson Laboratory (Bar Harbor, ME, USA) and maintained by cross-breeding male 5XFAD mice with female B6SJL/F1 mice to produce littermates. These 5XFAD mice harbor specific mutations in APP (V717I, I716V, and K670N/M671L) and PSEN1 (M146L and L286V), leading to robust Aβ overexpression. A cohort of 10 5XFAD mice was used in the study. Throughout the study, all mice were housed in a controlled environment with a 12/12 h light/dark cycle and provided ad libitum access to food and water. The animal sample size was determined using G*Power software (version 3.1).

All animal experiments were conducted in accordance with NIH guidelines (NIH Publications No. 8023, revised 1978) and were approved by the Institutional Animal Care and Use Committee of Dong-A University (DIACUC-approved-19-02). All experimental procedures were performed in accordance with the ARRIVE guidelines (https://arriveguidelines.org). Animals were randomly allocated to control and treatment groups using a random number table. Each animal was assigned a unique identification number, and the sequence of group allocation was determined according to the order of random numbers. This procedure ensured unbiased distribution across the groups. We did not control for potential confounders such as treatment order, measurement sequence, or cage location. However, to minimize experimental bias, a double-blind procedure was implemented, in which the individual administering the treatment and the experimenter conducting the behavioral assessments were different.

To minimize pain, suffering, and distress, all handling was performed gently, and the animals were monitored daily for changes in body weight, grooming, posture, or locomotor activity. No analgesics or anesthetics were required as no invasive procedures were performed. No unexpected adverse events occurred during the course of the study. Humane endpoints were established to reduce potential distress: animals showing signs of severe immobility, persistent abnormal posture, or inability to access food or water would have been removed from the study, but no animals met these criteria.

### 2.3. Tht Assay

For the aggregation assay, ThT was dissolved in DPBS, while Aβ_1–42_ was initially prepared at a concentration of 1 mM in DMSO before further dilution in DPBS. Curcumin and THB stocks were prepared in DMSO, with curcumin serving as the positive control. To evaluate the inhibition of Aβ_1–42_ aggregation, Aβ_1–42_ was co-incubated with various concentrations of THB (0, 1, 3, 10, or 30 μM) and ThT (45 μM) in black Eppendorf tubes for 24 h at 37 °C. The final Aβ_1–42_ concentration was maintained at 10 μM. Following incubation, 100 μL of each mixture was transferred to a black 96-well plate. Fluorescence intensities were measured in triplicate using a Synergy™ HTX multi-mode microplate reader (excitation: 485 nm; emission: 528 nm). In a separate disaggregation experiment, Aβ_1–42_ was pre-incubated for 24 h at 37 °C with continuous agitation to establish mature aggregates. These preformed fibrils were then treated with THB for an additional 24 h at 37 °C, and the resulting fluorescence levels were quantified according to the aforementioned procedure.

### 2.4. In Silico Molecular Docking Simulation

The crystal structures of the Aβ_1–42_ monomer (PDB ID: 6SZF) and fiber (PDB ID: 2BEG) were retrieved from the Protein Data Bank at resolutions ranging from 1.5 to 2.4 Å. The 3D coordinates for THB (CID: 5281553) were obtained from the PubChem database. Protein preparation, including atom type assignment, water molecule removal, and charge calculation, was performed using AutoDockTool 1.5.7. Docking simulations between THB and both Aβ_1–42_ monomers and fibers were executed using AutoDock Vina 1.1.2. The resulting binding poses were visualized and analyzed with PyMOL 2.5.0. Finally, pharmacophore analysis was conducted through the Ligplot+ program to determine the specific hydrogen bonding and van der Waals interactions between the compound and protein residues.

### 2.5. Cell Viability Assay

The neuroprotective effects of THB against Aβ_1–42_-induced toxicity were examined using the Neuro2a mouse neuroblastoma line (ATCC, Manassas, VA, USA). Cells were maintained in Minimum Essential Medium (WelGENE Inc., Gyeongsan, Republic of Korea) supplemented with 10% fetal bovine serum (FBS) and 1% penicillin/streptomycin at 37 °C in a CO_2_ incubator. For the assays, Neuro2a cells were seeded at a density of 2 × 10^4^ cells/mL and allowed to adhere for 24 h. The culture medium was then replaced with fresh medium containing 2% FBS and THB (0, 0.3, 1, 3, 10, 30 μM), followed by incubation with or without pre-aggregated Aβ_1–42_ (10 μM) for an additional 24 h. To assess cytotoxicity, the supernatant was collected and centrifuged at 1000 rpm for 2 min. The resulting supernatant was incubated with the LDH kit reagent (Sigma-Aldrich) for 20 min, and absorbance was recorded at 490 nm. For the cell viability measurement, the remaining cells were incubated with 0.5 mg/mL MTT solution for 30 min. After removing the solution, the formazan crystals were dissolved in DMSO and transferred to a 96-well plate for absorbance measurement at 540 nm.

### 2.6. Acute Hippocampal Slice Preparation and Electrophysiology

Electrophysiological measurements were conducted in artificial cerebrospinal fluid (aCSF) containing NaCl (124 mM), KCl (3 mM), NaHCO_3_ (26 mM), NaH_2_PO_4_ (1.25 mM), CaCl_2_ (2 mM), MgSO_4_ (1 mM), and D-glucose (10 mM). Following rapid isolation, the hippocampus was submerged in chilled aCSF. Coronal slices (400 μm thick) were prepared using a McIlwain tissue chopper and incubated in oxygenated aCSF (95% O_2_/5% CO_2_, 24–27 °C) for at least 1 h before being moved to the recording chamber. Field excitatory postsynaptic potentials (fEPSPs) were recorded from the stratum radiatum of area CA1, with a stimulating electrode placed on the Schaffer collateral–commissural pathway. To induce long-term potentiation (LTP), high-frequency stimulation (HFS, two trains of 100 pulses at 100 Hz) was applied after 20 min of baseline stability, and LTP was observed for 60 min post HFS. In the in vitro experiments, Aβ_1–42_ aggregates and THB were applied to the tissue slices for 2 h immediately before electrophysiological recording.

### 2.7. Aβ_1–42_ Preparation and Drug Administration

Drawing from a previous report that suggested the efficacy of 60 mg/kg THB in cerebral ischemia models [14], doses of 3 and 30 mg/kg were selected for the current in vivo experiments. Aβ_1–42_ was pre-incubated alone for 24 h at 37 °C with gentle agitation. These aggregates were then administered via intracerebroventricular injection into mice (coordinates: AP, −2.00 mm; mL, 0 mm; DV, −2.00 mm). THB (3 or 30 mg/kg, p.o.) was suspended in a 10% Tween 80 vehicle and administered daily for indicated days, starting 1 h after the aggregated Aβ_1–42_ (24 h incubation) injection. The passive avoidance test was initiated on the 6th day post-injection. For the 5XFAD model, THB was administered for 30 days beginning at 7 months of age. After the treatment period, brains were harvested and divided; one hemisphere was processed for electrophysiological analysis, and the other was used for immunohistochemistry.

### 2.8. Passive Avoidance Test

The passive avoidance task was implemented as a 2 d behavioral paradigm. Initially, each mouse was habituated to the light chamber for 10 s before the guillotine door opened. Entry into the dark chamber triggered a 0.5 mA shock, serving as an aversive stimulus. If the animal did not move within 60 s, it was excluded from the experiment. The following day, the time taken to re-enter the dark chamber was measured as an indicator of associative memory. Regarding the preparation of the Aβ_1–42_ model, aggregated Aβ_1–42_ (24 h incubation) was mixed with THB and incubated for an additional day before being injected into the brain ventricles. Behavioral training was scheduled 7 days after this procedure. In a separate assessment of THB’s standalone effects, mice were treated orally for a week before the trial, which utilized a lower shock intensity (0.25 mA). Shock sensitivity was further validated by observing behavioral responses such as vocalization, jumping, or running.

### 2.9. Brain Slice Preparation

To prepare the tissues for analysis, brains were removed and placed in 4% paraformaldehyde at 4 °C for overnight fixation. Cryoprotection was achieved by washing the fixed tissues in PBS and then soaking them in a 30% sucrose solution at 4 °C until they sank. For sectioning, a Leica freezing cryotome (Microm HM525) was used to cut the tissue into 30 μm serial slices. Finally, the sections were stored at 4 °C in a cryoprotection buffer containing 10% 0.2 M PB, 30% ethylene glycol, and 30% glycerin.

### 2.10. ThS Staining

To assess Aβ accumulation, ThS staining was performed. Six hippocampal slices were systematically selected (one out of every six sections relative to the bregma) and rinsed three times in PBS for 3 min each. The tissues were then incubated in a filtered 0.5% ThS solution (prepared in 50% EtOH) for 15 min. Following incubation, sections underwent sequential washes: three times in 50% EtOH and three times in PBS, each for 3 min. Finally, the stained sections were mounted onto microslides (Marienfeld, Germany) and secured with VectaMount AQ medium.

### 2.11. Immunohistochemistry

Immunohistochemistry followed a standard free-floating ABC protocol. Endogenous peroxidase activity was neutralized with 1% H_2_O_2_ before sections were incubated in a blocking solution (3% serum, 0.3% Triton X-100). Targeted proteins were detected using primary antibodies against GFAP, and Iba-1 (1:500 dilution). Secondary antibody labeling (1:200) and ABC reaction preceded a 3–10 min DAB development. Finally, sections underwent dehydration and xylene treatment prior to being mounted on silane-coated slides.

To quantify the immunopositive area, digital images of the stained hippocampal sections were captured using a microscope. The area of interest (AOI) was analyzed using ImageJ software (1.54g, National Institutes of Health, Bethesda, MD, USA). Specifically, the images were first converted to an 8-bit grayscale format to simplify the pixel data. A consistent threshold was then applied to each image to distinguish the immunopositive signals—such as Aβ plaques, GFAP-labeled astrocytes, or Iba-1-labeled microglia—from the background noise. The software then calculated the percentage of the area occupied by the positive staining relative to the total analyzed region. For each animal, the mean value from six representative sections was used for statistical comparison. All quantification procedures were performed by an investigator blinded to the experimental groups to ensure unbiased results.

### 2.12. Statistics

All experimental data were analyzed using one-way ANOVA followed by Tukey’s post hoc test for multiple comparisons. When only two groups were compared, Student’s *t*-test was used. If normality assumptions were not met, the Kruskal–Wallis test was applied. All analyses were performed by an investigator blinded to the experimental conditions to prevent bias. Results are presented as the mean ± SD, with statistical significance defined as *p* < 0.05.

## 3. Results

### 3.1. THB Reduced Toxic Aβ_1–42_ Aggregates

**Figure 1:**
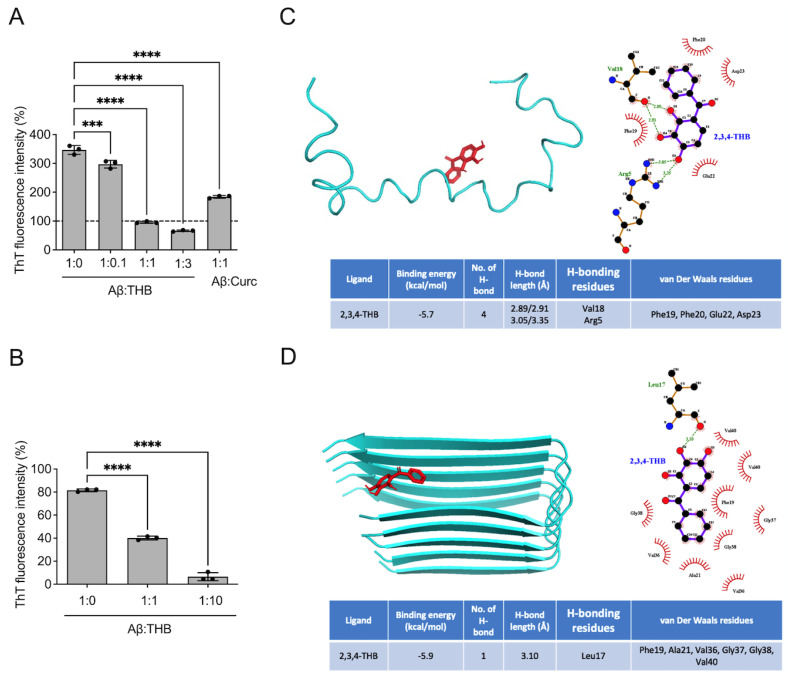
THB blocked Aβ_1–42_ aggregation and dissociated pre-aggregated Aβ_1–42_ aggregates. (**A**,**B**). Effect of THB on Aβ_1–42_ aggregates. (**A**). Anti-aggregation effect of THB. (**B**) Dissociation effect of THB. Data are presented as mean ± SD. *** *p* < 0.001. **** *p* < 0.0001. (**C**,**D**). Predicted binding poses from in silico molecular docking interactions of THB with Aβ_1–42_ fibril and Aβ_1–42_ monomer; surface view, interaction map, and hydrogen (dotted line in green) and hydrophobic bonding (red dashed semicircle) between drugs with Aβ_1–42_ fibril and monomer.

To investigate the effect of THB on the aggregation of Aβ_1–42_, we first aggregated Aβ_1–42_ (10 μM) in the presence of THB (1 or 10 μM) (Figure 1A). When Aβ_1–42_ was aggregated alone without THB, the ThT fluorescence intensity increased approximately three-fold compared to before aggregation (Figure 1A). However, when Aβ_1–42_ was aggregated with THB in a 1:1 ratio, the fluorescence intensity significantly decreased (*p* < 0.05, Figure 1A). Similarly, when curcumin, used as a positive control, was mixed with Aβ_1–42_ in a 1:1 ratio and aggregated, a significant decrease in fluorescence intensity was observed (*F*_4,10_ = 491.4, *p* < 0.05, Figure 1A). This indicates that THB inhibits the aggregation of Aβ_1–42_.

Next, to determine whether THB can disaggregate pre-formed Aβ_1–42_ aggregates, Aβ_1–42_ was first aggregated alone for 24 h, followed by the addition of THB and further incubation for another 24 h (Figure 1B). In the absence of THB, no change in fluorescence intensity was observed (Figure 1B). However, in the presence of THB, a significant decrease in fluorescence intensity was noted compared to before THB addition (*F*_2,6_ = 784.4, *p* < 0.05, Figure 1B). This suggests that THB can disaggregate pre-formed Aβ_1–42_ aggregates.

To predict potential binding modes, in silico docking simulations were conducted. Docking simulations predicted that THB could bind to the Aβ_1–42_ monomer with minimum energies of −5.7 kcal/mol (Figure 1C). In addition, THB could adopt poses compatible with hydrogen bonding with the Val18 and Arg5 and hydrophobic interactions with surrounding residues such as Phe19, Phe20, Glu22 and Asp22 (Figure 1C). Moreover, two hydrogen bonds between THB and Val18 and Arg5 were predicted, with bonding distances of 2.89/2.91 Å and 3.05/3.35 Å, respectively (Figure 1C). Dockinh simulations predicted that THB could bind to Aβ_1–42_ fibrils with minimum energies of −5.9 kcal/mol (Figure 1D). Furthermore, THB formed hydrogen bonds with Leu17, and hydrophobic interactions with surrounding residues such as Phe19, Ala21, Val36, Gly37, Gly38 and Val40 (Figure 1D). The interaction between THB and Leu17 occurred with a predicted bond distance of 3.10 Å.

### 3.2. THB Blocked Aβ_1–42_ Aggregate-Induced Cytotoxicity in Neuro2a Cells

**Figure 2:**
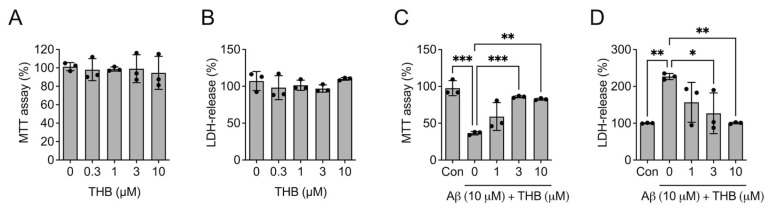
THB attenuated Aβ_1–42_ cytotoxicity in neuro2a cells. (**A**,**B**). Effect of THB on cell viability. Neuro2a cells were incubated with THB (0.3, 1, 3 or 10 μM) for 24 h. A. Effect of THB on neuro2a cell viability in MTT assay. (**B**) Effect of THB on cytotoxicity in LDH-release assay. (**C**,**D**). Effect of THB on Aβ_1–42_-induced neuro2a cell death. Neuro2a cells were incubated with THB (0.3, 1, 3 or 10 μM) for 1 h and then incubated with THB + Aβ_1–42_ (10 μM) for 24 h. (**C**) Effect of THB on neuro2a cell viability in MTT assay. D. Effect of THB on cytotoxicity in LDH-release assay. Data are presented as mean ± SD. * *p* < 0.05. ** *p* < 0.01. *** *p* < 0.001.

To assess whether the modulation of Aβ_1–42_ aggregation by THB could reduce Aβ_1–42_ toxicity, the effect of THB alone on cell viability was first assessed. THB did not affect MTT level (*F*_4,10_ = 0.123. *p* > 0.05, *n* = 3, Figure 2A) and LDH release (*F*_4,10_ = 0.942, *p* > 0.05, *n* = 3, Figure 2B), indicating that THB alone did not exert cytotoxic effects under these experimental conditions. Next, Aβ_1–42_ and THB were co-incubated with Neuro2a cells for 24 h. In the group where Aβ_1–42_ was incubated alone, a significant decrease in MTT (*F*_4,10_ = 18.95, *p* < 0.05, *n* = 3, Figure 2C) and an increase in LDH (*F*_4,10_ = 6.868, *p* < 0.05, *n* = 3, Figure 2D) were observed, confirming the neurotoxicity of Aβ_1–42_. However, in the group treated with a mixture of THB and Aβ_1–42_, these changes were suppressed (Figure 2C,D). This indicates that THB reduces the toxicity of Aβ_1–42_, likely by decreasing the formation of toxic Aβ_1–42_ aggregates.

### 3.3. THB Blocked LTP Impairment in the Hippocampus

**Figure 3:**
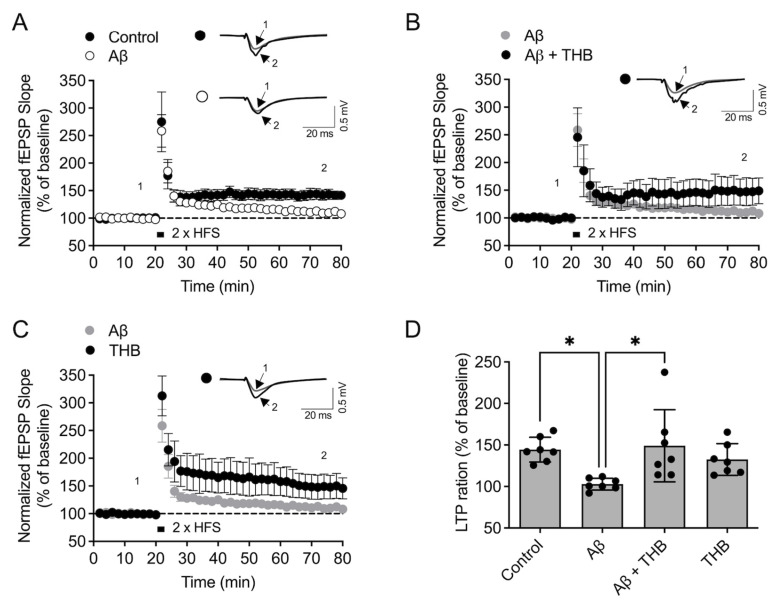
THB attenuated Aβ_1–42_ synaptotoxicity in the hippocampus of male mice. Acute hippocampal slices were incubated with THB for 30 min and then incubated with THB + Aβ_1–42_ for 2 h. (**A**–**C**). Changes in normalized fEPSP slope over time in the control and Aβ_1–42_-treated group (**A**), Aβ_1–42_ + THB-treated group (**B**), and THB-treated group (**C**) with representative images of single traces. Point 1 indicates the time point 5 min before high-frequency stimulation (HFS), and Point 2 indicates the time point 5 min before the end of the recording. (**D**) The average value of fEPSP over the last 5 min. Data are presented as the mean ± SD. * *p* < 0.05.

Memory impairments in AD are closely related to the decline in synaptic function caused by Aβ_1–42_ aggregates. Therefore, we next examined the effect of THB on Aβ_1–42_ aggregate-induced LTP impairment. In hippocampal slices treated with Aβ_1–42_ aggregates for 2 h, significantly lower LTP was observed compared to slices treated with the vehicle for 2 h (*F*_3,24_ = 4.778, *p* < 0.05, *n* = 7/group, Figure 3A,D). This indicates that Aβ_1–42_ aggregates induced a reduction in LTP. In hippocampal slices treated with both Aβ_1–42_ and THB (10 μM) simultaneously, no significant difference in LTP was observed compared to the control group (Figure 3B), and LTP was significantly higher than in the group treated with Aβ_1–42_ alone (Figure 3B,D). In hippocampal tissues treated with THB alone, LTP levels were similar to those of the control group (Figure 3C,D), indicating that THB itself does not affect LTP. These results suggest that THB can mitigate the LTP inhibition caused by Aβ_1–42_, likely due to THB’s ability to inhibit Aβ_1–42_ aggregation.

### 3.4. THB Blocked Aβ_1–42_ Aggregate-Induced Memory Impairments

**Figure 4:**
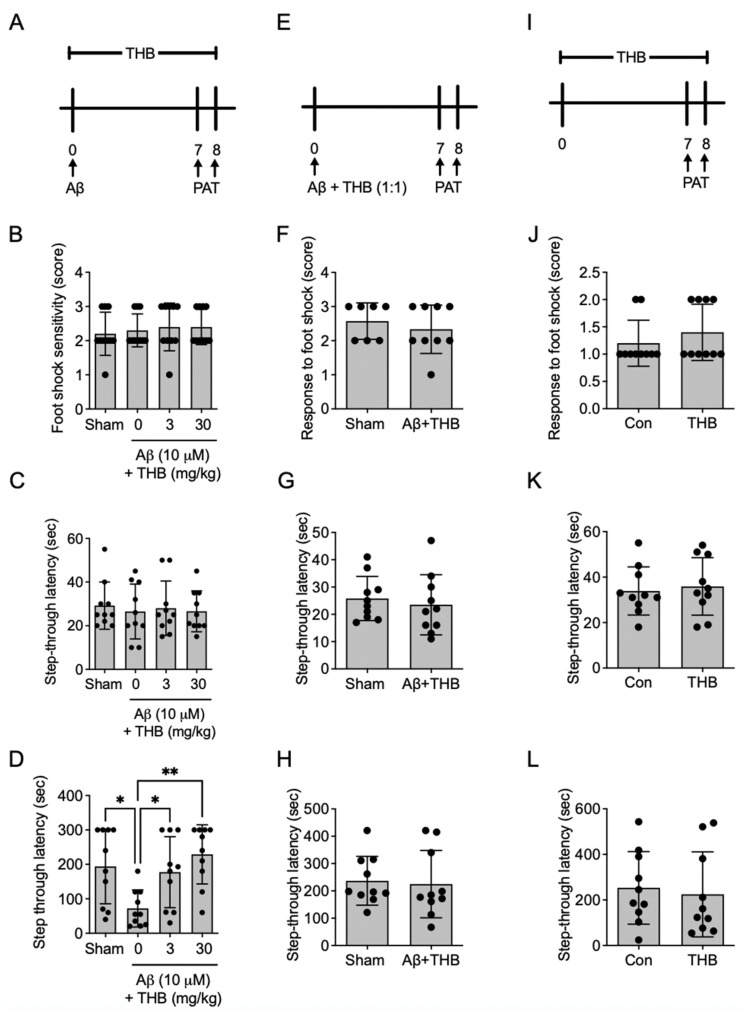
THB ameliorated Aβ_1–42_ aggregate-induced memory impairment in male mice. (**A**–**D**). The effect of THB on Aβ_1–42_ aggregate-induced memory impairment in male mice. (**A**) Experimental schedule. In experimental animals, THB was orally administered once daily. After 7 days, a passive avoidance test (PAT) was conducted. In the training trial of PAT, the response to foot shock (**B**) and the latency to move to the dark chamber (**C**) were measured, along with the latency to move to the dark chamber during the test trial (**D**). (**E**–**H**) The effects of THB on toxic Aβ_1–42_ production. After co-incubating Aβ_1–42_ with THB for 24 h, the mixture was administered into the lateral ventricles of the experimental animals. After seven days, a passive avoidance test was conducted (**E**). In the training trial of PAT, the response to foot shock (**F**) and the latency to move to the dark chamber (**G**) were measured, along with the latency to move to the dark chamber during the test trial (**H**). (**I**–**L**) The effect of THB on memory. THB was orally administered to the experimental animals once daily for one week, followed by a passive avoidance test (**I**). In the training trial of PAT, the response to foot shock during the training trial (**J**) and the latency to move to the dark chamber (**K**) were measured, along with the latency to move to the dark chamber during the test trial (**L**). Data are presented as the mean ± SD. * *p* < 0.05. ** *p* < 0.01.

Next, to confirm whether THB is effective in mitigating memory impairment induced by Aβ_1–42_ aggregates, we conducted a passive avoidance test after orally administering THB for one week to a memory impairment model created by injecting Aβ_1–42_ aggregates into the brain lateral ventricle (Figure 4A). The administration of Aβ_1–42_ aggregates and THB did not affect sensitivity to foot shock (*F*_3,36_ = 0.264, *p* > 0.05, *n* = 10/group, Figure 4B) or latency time measured during the training trial (*F*_3,36_ = 0.264, *p* > 0.05, *n* = 10/group, Figure 4C). However, in the test trial, the group injected with Aβ_1–42_ aggregates and given only the vehicle showed significantly lower latency time compared to the sham group (*F*_3,36_ = 5.584, *p* < 0.05, *n* = 10/group, Figure 4D), indicating that Aβ_1–42_ aggregates impaired memory. In contrast, the group treated with THB (3 or 30 mg/kg) after Aβ_1–42_ aggregates injection showed a dose-dependent increase in latency time (*p* < 0.05, Figure 4D), suggesting that THB can improve memory impairment caused by Aβ_1–42_ aggregates.

Next, to determine whether THB reduced toxic Aβ_1–42_ species, Aβ_1–42_ was incubated with THB for 24 h and then injected into the mouse brain lateral ventricle. Seven days later, a passive avoidance test was conducted (Figure 4E). No changes in sensitivity to foot shock (*t*_18_ = 0.447, *n* = 10/group, *p* > 0.05, Figure 4F) or latency time during the training trial (*t*_18_ = 0.532, *n* = 10/group, *p* > 0.05, Figure 4G) were observed in any group, and no changes in latency time were observed during the test trial (*t*_18_ = 0.251, *n* = 10/group, *p* > 0.05, Figure 4H). This indicates that THB dissociated Aβ_1–42_ aggregates to a level that could not induce memory impairment.

Finally, to verify whether THB itself affects memory, THB was orally administered for seven days, followed by a passive avoidance test (Figure 4I). The results showed that THB had no effect on sensitivity to foot shock (*t*_18_ = 0.948, *n* = 10/group, *p* > 0.05, Figure 4J) or latency time during both training (*t*_18_ = 0.384, *n* = 10/group, *p* > 0.05, Figure 4K) and test trials (*t*_18_ = 0.367, *n* = 10/group, *p* > 0.05, Figure 4L). These findings suggest that THB inhibits the memory-impairing effects of Aβ_1–42_ aggregates, likely due to its ability to inhibit Aβ_1–42_ aggregation and dissociate Aβ_1–42_ aggregates.

### 3.5. THB Reduced Synaptic Deficit in the Hippocampus of 5XFAD Male Mice

**Figure 5:**
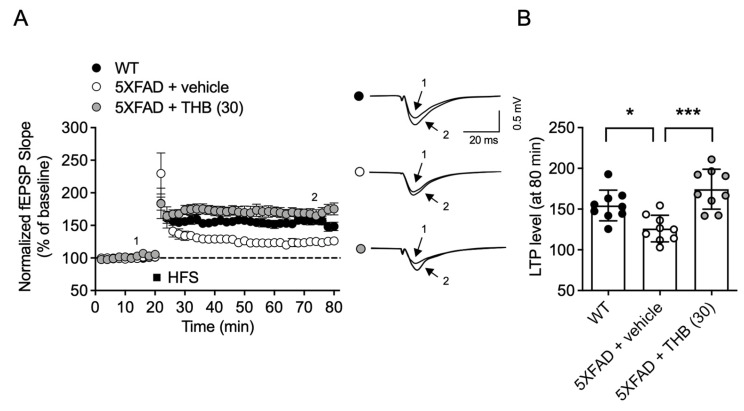
THB ameliorated LTP deficit in the hippocampus of 5XFAD mice. 5XFAD male mice were treated with THB (30 mg/kg, p.o.) once a day for 1 month. Acute hippocampal slices were prepared 1 day after the last THB administration. (**A**) Changes in normalized fEPSP slope over time with representative images of single traces. (**B**) The average value of fEPSP over the last 5 min. Data are presented as the mean ± SD. * *p* < 0.05. *** *p* < 0.001.

Finally, to verify the effectiveness of THB in another AD animal model, we administered THB orally for one month to 8-month-old 5XFAD mice and then extracted their brains to measure LTP. As previously reported, the hippocampus of 5XFAD mice exhibited significantly lower LTP compared to that of wild-type mice (*F*_2,24_ = 13.02, *p* < 0.05, *n* = 9/group, Figure 5A,B). However, the hippocampus of 5XFAD mice treated with THB for one month showed LTP levels similar to those of WT mice (Figure 5A,B). This indicates that THB can mitigate brain function decline in the genetic AD model, 5XFAD mice.

### 3.6. THB Reduced AD-like Pathology in 5XFAD Male Mice

**Figure 6:**
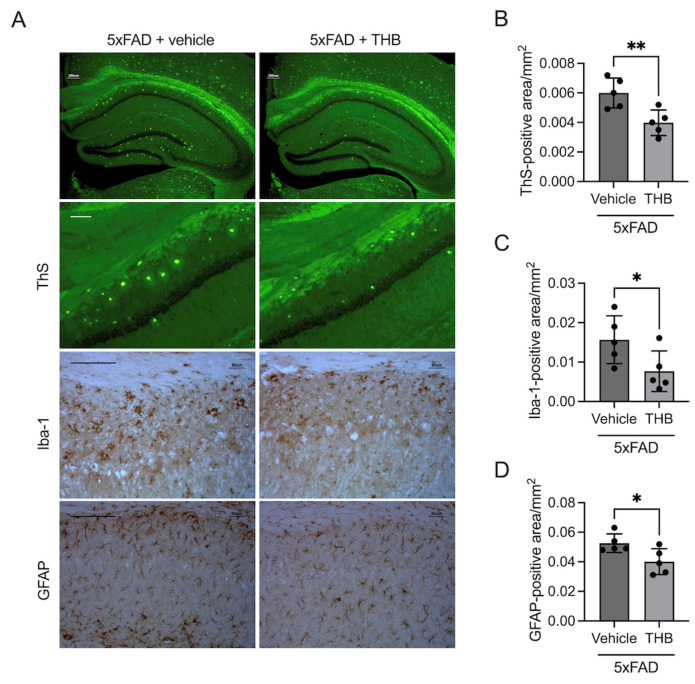
THB reduces Aβ pathology and neuroinflammation in 5XFAD mice. (**A**) Representative hippocampal images showing ThS-stained Aβ plaques and IHC-stained Iba-1 (microglia) and GFAP (astrocytes). (**B**–**D**) Quantification of (**B**) ThS, (**C**) Iba-1, and (**D**) GFAP-positive areas using ImageJ. 5XFAD mice (7 months old) received vehicle or THB (30 mg/kg, p.o.) daily for 30 days. Data are mean ± SD (*n* = 5); * *p* < 0.05 and ** *p* < 0.01 via Student’s *t*-test. Bar = 200 μm.

To evaluate the therapeutic potential of THB on Aβ pathology, 7-month-old 5XFAD mice were administered THB for 30 days. Histological analysis via ThS staining revealed a significant decrease in Aβ plaque density within the THB-treated group compared to the vehicle-treated control (*t*_8_ = 3.393, *p* < 0.05, *n* = 5/group, Figure 6A,B). Furthermore, THB treatment effectively attenuated neuroinflammatory markers, as evidenced by a substantial reduction in Iba-1-positive (microglia, *t*_8_ = 2.248, *p* < 0.05, *n* = 5/group, Figure 6A,C) and GFAP-positive (astrocyte, *t*_8_ = 2.597, *p* < 0.05, *n* = 5/group, Figure 6A,D) areas. These findings demonstrate that THB is effective in alleviating Aβ plaque accumulation and the subsequent neuroinflammatory response.

## 4. Discussion

In this study, we demonstrated that THB inhibits Aβ_1–42_ aggregation in a concentration-dependent manner and reduces aggregate levels by promoting their disassembly. THB also attenuated Aβ_1–42_-induced cytotoxicity in Neuro2a cells, restored hippocampal LTP in acute slices, and improved memory impairment in an Aβ_1–42_ aggregate-induced model. In addition, THB reduced inflammatory marker expression and immune cell activation associated with Aβ aggregation. Finally, THB ameliorated LTP deficits in the hippocampus of 5XFAD male mice, a genetically engineered model of AD. Collectively, these findings suggest that THB improves memory deficits in AD models, likely through inhibition and disassembly of Aβ aggregates, attenuation of cytotoxicity, and suppression of neuroinflammatory responses.

Abnormal aggregation of Aβ, although structural details remain debated, is generally characterized by an increase in β-sheet content [10]. The resulting aggregates are considered a hallmark of AD pathology. Accordingly, therapeutic strategies targeting Aβ—such as inhibition of β-site amyloid precursor protein cleaving enzyme 1, γ-secretase modulation, or stabilization of Aβ monomers—have been actively pursued [15,16]. However, successful clinical outcomes remain limited. This may be attributable, in part, to the physiological roles of enzymes involved in Aβ production, which are essential for normal brain function [17]. Moreover, emerging evidence indicates that Aβ monomers contribute to synaptic regulation and memory formation, suggesting that indiscriminate suppression of Aβ production may impair normal neuronal function [18]. Therefore, more selective approaches beyond reducing overall Aβ production are needed.

While Aβ monomers are generally considered non-toxic, aggregated forms exert multiple deleterious effects [10]. Thus, preventing Aβ aggregation while preserving monomeric function may represent a more precise therapeutic strategy. The Aβ_16–22_ region (KLVFFAE) has been identified as a critical fibrillogenic segment with high β-sheet-forming propensity [19,20]. Among several macrocyclic peptides incorporating Aβ-derived segments, constructs containing the 16–22 sequence have been shown to inhibit aggregation and reduce cytotoxicity [21]. In the present study, molecular docking analysis predicted that THB forms hydrogen bonds with Val18 and Arg5 and engages in hydrophobic interactions with residues including Phe19, Phe20, Glu22, and Asp22. These findings suggest that THB may interact with residues within or near the Aβ_16–22_ region, potentially contributing to its anti-aggregation activity. However, further biophysical studies are required to confirm these interactions experimentally.

In patients with established amyloid deposition, inhibiting further aggregation alone may have limited therapeutic impact. Indeed, recent antibody-based therapies that remove Aβ aggregates have demonstrated only modest efficacy and are associated with adverse effects [22]. Therefore, early intervention to prevent or limit Aβ aggregation before extensive deposition occurs may represent a more effective strategy. However, because Aβ accumulation begins decades before clinical diagnosis [10], long-term use of costly biologics in asymptomatic individuals is impractical. In this context, readily accessible small molecules that inhibit Aβ aggregation may provide a conceptual foundation for preventive approaches, although substantial optimization and safety evaluation will be required.

THB is an inexpensive compound that is widely used commercially, particularly in cosmetic formulations for ultraviolet protection. Owing to its structural features, it is expected to possess diverse biological activities [23,24]. Previous studies have reported that THB inhibits aggregation of both Aβ and tau proteins [25]. In our study, structurally related benzophenone derivatives exhibited similar anti-aggregation effects. However, benzophenone compounds have been associated with endocrine-disrupting activity, which complicates their development as therapeutic agents. Notably, among these compounds, THB has been reported to exhibit relatively weaker hormone-disrupting effects [26]. Thus, THB may serve as a useful lead structure for the development of safer derivatives capable of inhibiting Aβ aggregation.

Importantly, the present study extends previous in vitro findings by demonstrating in vivo reductions in amyloid pathology. Thioflavin S staining in 5XFAD mice revealed a significant decrease in fibrillar Aβ plaque burden following THB treatment. This histological evidence indicates that THB can attenuate established amyloid pathology in a transgenic AD model. The reduction in plaque load is consistent with the dual ability of THB to inhibit aggregation and promote the disassembly of pre-existing fibrils, suggesting that prolonged administration may shift the equilibrium away from pathogenic Aβ assemblies.

Consistent with plaque reduction, THB significantly attenuated neuroinflammatory responses, as evidenced by decreased Iba-1–positive microglial activation and reduced GFAP-positive astrocytic reactivity in the hippocampus of 5XFAD mice. Neuroinflammation is increasingly recognized as a critical driver of synaptic dysfunction and disease progression in AD [27,28]. Microglia, in particular, respond rapidly to Aβ deposition and release pro-inflammatory mediators that exacerbate neuronal injury. The observed reduction in glial activation following THB treatment may therefore reflect decreased Aβ burden and reduced inflammatory signaling.

The functional relevance of these pathological changes is supported by the restoration of hippocampal LTP in THB-treated 5XFAD mice. Because synaptic plasticity deficits strongly correlate with cognitive impairment in AD, recovery of LTP provides evidence that THB-mediated reductions in Aβ aggregation and neuroinflammation translate into improved neuronal function. Importantly, THB did not alter synaptic plasticity in normal hippocampal tissue, suggesting that its beneficial effects are unlikely due to nonspecific synaptic enhancement but rather to mitigation of Aβ-driven pathology.

Although THB itself is predicted to have limited blood–brain barrier permeability due to its multiple phenolic hydroxyl groups, in silico ADME analysis suggested that O-methylated metabolites of THB may possess physicochemical properties favorable for BBB penetration. These computational predictions are hypothesis-generating and require experimental validation. Direct pharmacokinetic studies assessing plasma and brain concentrations of THB and its metabolites will be necessary to determine whether central exposure contributes to the observed in vivo effects (Supplementary Figure S1).

The therapeutic doses of THB were selected based on previous efficacy observed in a cerebral ischemia model. However, ischemic injury and AD differ substantially in blood–brain barrier integrity and pathological mechanisms. Therefore, extrapolation of dosing across disease models has inherent limitations. Comprehensive pharmacokinetic and dose–response studies will be required to optimize therapeutic strategies for AD-specific applications.

No significant changes in body weight were observed during THB treatment in either the Aβ-injected or 5XFAD models (Supplementary Figure S2), and daily monitoring revealed no abnormal behavioral signs, including reduced grooming, impaired locomotion, or lethargy, indicating the absence of overt systemic toxicity at the tested doses in our mammalian models. However, despite the pronounced anti-amyloidogenic and neuroprotective effects demonstrated in this study, recent toxicological evidence suggests that THB may exert adverse effects under certain exposure conditions. In a zebrafish-based model, THB exposure induced cardiac and neurological toxicity, such as pericardial edema, impaired neuronal differentiation, and behavioral deficits, which were associated with the dysregulation of energy metabolism-related pathways involving pgam1a and pgk1 [29]. These findings underscore the importance of further safety evaluation across multiple biological systems. Rather than diminishing its therapeutic relevance, such considerations highlight the need for structural refinement to preserve anti-amyloidogenic activity while minimizing potential adverse effects.

The present study was conducted exclusively in male mice to reduce hormonal variability. However, AD exhibits sex-specific differences in prevalence and pathology, with female 5XFAD mice often demonstrating accelerated amyloid deposition. Future studies should therefore assess the efficacy and safety of THB in female models to determine potential sex-dependent responses.

Although a formal dose–response analysis was not performed, two doses were selected to represent a preliminary therapeutic range. While a dose-dependent trend was observed in behavioral outcomes, future studies incorporating additional dose levels and pharmacokinetic measurements will be necessary to define the optimal therapeutic window.

In summary, the present study demonstrates that THB inhibits toxic Aβ_1–42_ aggregation, reduces amyloid plaque burden, attenuates neuroinflammatory responses, and improves synaptic plasticity and memory performance in experimental models of AD. By targeting pathogenic Aβ assemblies rather than global Aβ production, THB provides a promising lead scaffold for the development of preventive or disease-modifying therapeutic strategies for AD.

## Data Availability

The original contributions presented in this study are included in the article/Supplementary Materials. Further inquiries can be directed to the corresponding author.

## Supplementary Materials

The following supporting information can be downloaded at: https://www.mdpi.com/article/10.3390/pharmaceutics18030320/s1, Figure S1: In silico prediction of blood–brain barrier (BBB) permeability of THB and its O-methylated metabolite using SwissADME. Figure S2. Effect of THB on body weight change in experimental mouse models.

## Abbreviations

The following abbreviations are used in this manuscript:

AD	Alzheimer’s disease
Aβ	Amyloid β
THB	2,3,4-trihydroxybenzophenone
LTP	Long-term potentiation
NMDA	N-methyl-D-aspartate
NFTs	Tau neurofibrillary tangles
MTT	3-(4,5-dimethylthiazol-2-yl)-2,5-diphenylatetrazolium bromide
ThT	Thioflavin T
ThS	Thioflavin S
FBS	Fetal bovine serum
aCSF	Artificial cerebrospinal fluid
fEPSP	Field excitatory postsynaptic potential
HFS	High-frequency stimulation
