# Supplementary material for: 2,3,4-Trihydroxybenzophenone Disassembles Amyloid β Aggregates and Ameliorates Synaptic Deficits
Source: Pharmaceutics. 2026 Mar 2;18(3):320. doi: 10.3390/pharmaceutics18030320 (PMC13028855; doi:10.3390/pharmaceutics18030320)
Supplement: Supplementary file 1 [file pharmaceutics-18-00320-s001.zip › Figure S1 2.pdf]

Figure S1

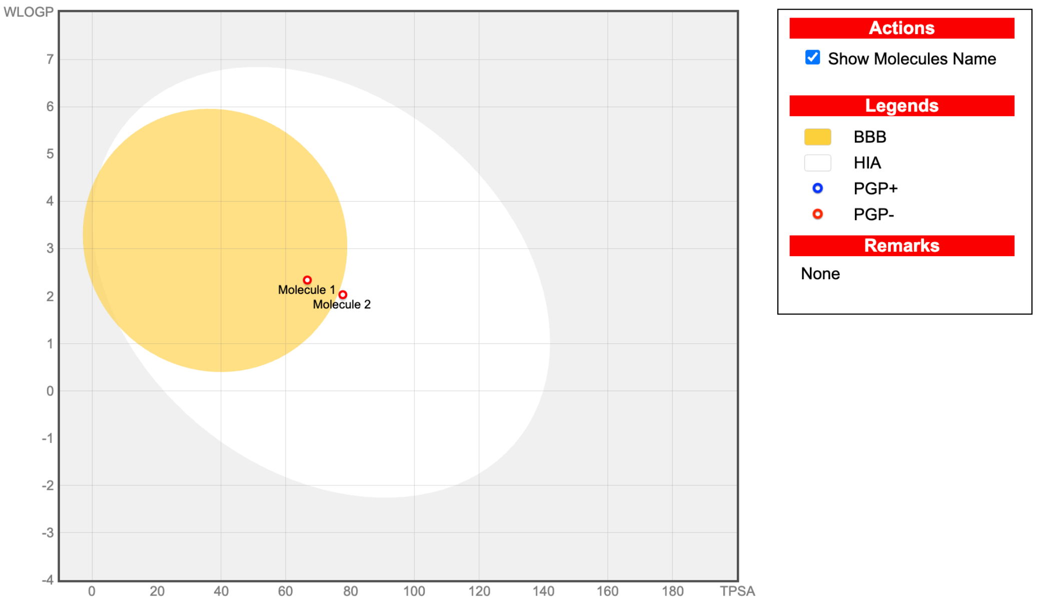

Figure S1. In silico prediction of blood–brain barrier (BBB) permeability of THB and its O-methylated metabolite using SwissADME. BOILED-Egg plot representing the re-lationship between lipophilicity (WLOGP) and topological polar surface area (TPSA). The yellow region indicates the physicochemical space associated with a high probability of BBB permeation, whereas the white region corresponds to high human intestinal ab-sorption (HIA). Molecule 1 represents an O-methylated metabolite of THB, and Molecule 2 represents the parent compound THB. Both molecules are predicted as P-glycoprotein non-substrates (PGP−). The O-methylated metabolite (Molecule 1) is located within the BBB-permeant region, suggesting that metabolic O-methylation may enhance the like-lihood of BBB penetration compared to the parent compound.

Figure S2

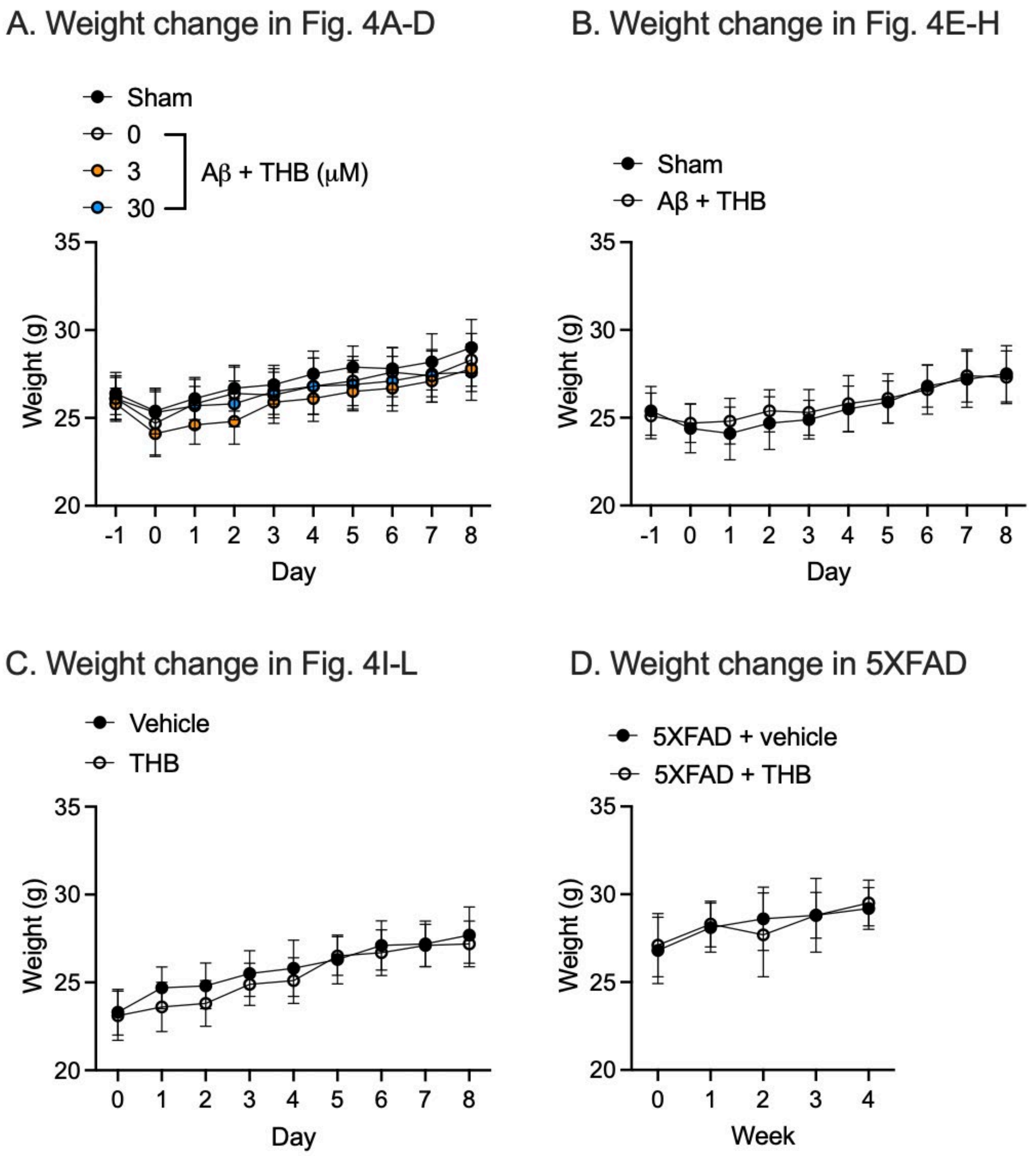

Figure S2. Effect of THB on body weight change in experimental mouse models. (A) Body weight change in mice shown in Fig. 4A–D. Mice were treated with Aβ (i.c.v.) in the presence or absence of THB (0, 3, or 30 mg/kg). Sham controls are indicated. Body weight (g) was measured daily from day −1 to day 8. (B) Body weight change in mice shown in Fig. 4E–H. Sham and Aβ + THB groups were monitored daily from day −1 to day 8. (C) Body weight change in mice shown in Fig. 4I–L. Mice received vehicle or THB treatment, and body weight was recorded daily from day 0 to day 8. (D) Body weight change in 5XFAD mice treated with vehicle or THB. Body weight was measured

weekly from week 0 to week 4. Data are presented as mean ± SEM. No significant differences in body weight were observed among groups over the experimental period.
